# EFFECTIVENESS OF THE “SELF-PROGRAM” IN DUTCH NURSING HOME CARE: RESULTS OF A MULTICENTER CLUSTER-RANDOMIZED TRIAL

**DOI:** 10.1093/geroni/igad104.1117

**Published:** 2023-12-21

**Authors:** Stan Vluggen, Silke Metzelthin, Sandra Zwakhalen, Michel Bleijlevens, Janneke M de Man-van de van de van Ginkel, Getty Huisman - De Waal

**Affiliations:** Zuyd Hogeschool University of Applied Sciences, Kerkrade, Limburg, Netherlands; Maastricht University, Care and Public Health Research Institute, Maastricht, Limburg, Netherlands; Maastricht University, Maastricht, Limburg, Netherlands; Maastricht University, Maastricht, Limburg, Netherlands; Leiden University Medical Center, Leiden, Zuid-Holland, Netherlands; Radboud University Medical Center, Nijmegen, Gelderland, Netherlands

## Abstract

For frail older people it is evident to maintain their functional abilities and independence. Nursing staff are in a key position to encourage older people’s independence and stimulate their engagement in functional activity. However, nurses tend to take over tasks frequently thereby depriving older people’s remaining abilities. Clearly nurses need support to optimize their activity encouragement behavior. The Function-Focused Care based ‘SELF-program’ was developed based on lessons learned and implications from previous programs. SELF is an interactive, tailored, holistic and theory-grounded training program consisting of 7 seven sessions spread over a period of 3 months which aims to improve nurses’ activity encouragement behavior and in turn client’s self-reliance in daily activities. The program was subjected to a cluster-randomized trial in Dutch nursing home care which aimed to evaluate the program’s effectiveness. The MAINtAIN and GARS-5 questionnaires were applied to assess the effectiveness on nurses’ activity encouragement behavior and clients’ self-reliance. 28 wards from three care organizations across the Netherlands were recruited and randomized to the SELF-program or care as usual. 287 nurses and 241 geriatric clients participated. Measurements took place at baseline, directly after implementing the SELF-program (three months) and 9 months after baseline. Mixed linear regression demonstrated that nurses’ activity encouragement behavior significantly improved (p=.004; ES=.05). At client level, no effects were found (p=.09; ES=.13). However, a trend towards slower decline in self-reliance was observed for those clients allocated to the SELF-program condition. The SELF-program was effective in improving nurses’ behavior, however, effects were not translated to client populations.

